# What Might it Mean for Political Theory to Be More ‘Realistic’?

**DOI:** 10.1007/s11406-016-9799-3

**Published:** 2017-01-17

**Authors:** John Horton

**Affiliations:** School of Politics, Philosophy, International Relations and Environment (SPIRE), Keele University, Staffordshire, ST5 5BG UK

**Keywords:** Ideal theory, Interpretative realism, Liberal moralism, Normativity, Prescriptive realism

## Abstract

This paper explores two different versions of ‘the realist turn’ in recent political theory. It begins by setting out two principal realist criticisms of liberal moralism: that it is both descriptively and normatively inadequate. It then pursues the second criticism by arguing that there are two fundamentally different responses among realists to the alleged normative inadequacy of ideal theory. First, prescriptive realists argue that the aim of realism is to make political theory more normatively adequate by making it more realistic. Interpretative realists, on the other hand, argue that realist theorising should detach itself from such an aspiration, and instead aim at theoretical understanding rather than normative prescription. After some further elaboration of what interpretative realism might look like, it is acknowledged that both approaches still need to address the question of political normativity.

One of the great pleasures offered by watching the American TV series, *The West Wing*, is that it presents a picture of political life as reasonably close to how many ordinary people, especially those who are moderately left-leaning, would like it to be. A wise and principled US President, occasionally willing to engage in some devious behaviour, but only in a good cause and almost always with virtuous motives, is supported by a mostly morally decent political staff. The latter may be rather more inclined to stray from the path of righteousness, but when that happens they are also mainly moved by understandable human weaknesses or a misguided sense of duty or loyalty, and usually corrected by the sagacious President. That is TV. Unfortunately, as we all know, even with a pretty honourable man like Obama as President, actual US politics bears little relationship to the fictional world of *The West Wing*; if anything *House of Cards* might be a more accurate guide. And this is not because American politics is uniquely, or even especially, deficient in virtue: governmental politics everywhere is, to a greater or lesser extent, similarly flawed if judged by such standards.

Much of contemporary normative political theory^1^ in the English-speaking world, although by no means all of it, but often including the most prestigious work, is, or so I believe, rather like *The West Wing* only more so, in that it presents us with a highly idealised image of how political life should be that is basically at best an edifying fantasy. Normative political theory, too, portrays a world very largely governed by moral principles and to an even greater extent ignores the motivational and practical complexities of political life. Of course, a crucial difference might be thought to be that normative political theory is *normative*, which means that it is about ideals and prescription rather than mere description. It is about how politics ought to be, not about how it is; and, therefore, it is simply a mistake to confuse prescription with description. And so it is, but even normative political theory surely needs to be firmly rooted in an understanding of human experience and political possibility that is genuinely plausible, if it is to have something serious to say about *politics*, as we know it. It is on questions about the normativity of political theory, about the viability of its prescriptive status and whether there could be any other kind of worthwhile political theory that the ensuing discussion will primarily focus.

In particular, I shall consider some issues that arise ultimately from a concern about the *point* of political theory, but are most obviously reflected in a concern with the form in which it is practiced. It is, therefore, a somewhat introspective enquiry, although not I hope one without wider significance and interest. At its heart lie questions about what we should expect from good political theory; about how we should assess the value or worth of political theory; and about the relationship between political theory and political practice. These are questions to which no one with any interest in political theory can honestly be entirely indifferent, even if they may not much excite anyone else. I locate this enquiry within a current ‘debate’, if that is quite the right word, which has become increasingly prominent in the last few years, between proponents of what is sometimes called ‘ideal theory’ and their ‘realist’ critics, although it is also somewhat wider than that. In describing it as ‘current’ however I do not intend to suggest that the issues are new. Rather, they have arisen in relation to reflective thinking about political life more or less since what we now describe as political theory first began; it is, for instance, one of the issues that can be seen to divide Plato’s Socrates from some of his interlocutors in the *Republic*. And it is something that has recurred from time-to-time, in various guises, throughout the history of political theorising. However, I shall only be concerned with the way that these issues arise and are played out in contemporary political theory. This should certainly not, though, be taken as an expression of either hostility or dismissiveness towards the study of the history of political theory. In fact, for what it is worth, and pretty much like all realists and their sympathisers, I believe that political theorising would benefit from greater sensitivity to history generally, and to its own history in particular.

It should also be made clear from the start that this discussion is primarily exploratory and suggestive, and neither has nor claims a compelling conclusion. There are many questions about what I shall go on to say that in truth I do not know how best to answer; although I am thoroughly convinced that they are worth taking seriously. Trying to show that is so is one of my principal aims. Moreover, while there is indeed no tightly argued conclusion, it would be disingenuous to pretend that these reflections do not at least incline in a certain direction. I do want to encourage a rather different way of thinking about political theory. My sympathies, as will no doubt be evident, lie firmly with the realist camp. However, as will shortly be seen, I depart sharply from many of my fellow-travellers on one important issue. The schism that results from this difference about whether political theory should aspire to be action-guiding is quite fundamental to how realists conceive political theory and how they think it should be pursued. Probably the most distinctive part of the discussion is the exploration of that fissure.

The ‘realist’ critique – and the sense of ‘realism’ intended here is to be understood in its naïve or ordinary meaning, not as a metaphysical view about the nature of reality nor even as the concept is used in international relations theory – is primarily directed at what has been variously labelled ‘high liberalism’, ‘political moralism’ or ‘liberal moralism’ and indeed most forms of ‘ideal theory’: basically, the targets are political philosophers such as Rawls, Rawlsians, their friendly critics and others who work in a similar genre (although, in fairness, it could certainly be extended to much post-structuralist theorizing, too). The critique is loosely associated with Americans such as Judith Shklar and Bonnie Honig, but is found particularly strongly expressed by philosophers and theorists working in Britain (even if its exponents are not always British), such as Bernard Williams, John Gray, Stuart Hampshire, Raymond Geuss, Chantal Mouffe. Glen Newey, Mark Philp, Matt Sleat and, one of the earliest ploughers of this furrow, John Dunn.^2^ As will be obvious to anyone familiar with their work, these thinkers are very different in a great many respects and certainly do not constitute a school or even share anything that could properly be called a common position. Some of them might not even recognise the characterisation of their work as (even in part) realist. They are perhaps best regarded, in the words of William Galston, who has provided us with probably the best overview of the realist critique, as ‘a kind of community stew where everyone throws something different into the pot’ (Galston 2010: 386). But there is also, as he adds, ‘a theme or sentiment that unites realists at the threshold – a belief that high liberalism represents a desire to evade, displace, or escape from politics’ (Galston 2010: 386). They are all strongly critical of a form of political theory in which ‘the political’ becomes denigrated, repressed or effaced.

I do not here intend to rehearse the realist critique in any detail, but a short account of what seems to me central to it is perhaps necessary for an appreciation of what follows. There are, I suggest, two related but distinguishable broad lines of criticism that lie at the heart of the realist critique. First, there is the complaint that the conception of politics at work in liberal moralism, as I shall henceforth call their target, lacks *descriptive* adequacy. Secondly, there is the objection that it is utopian in a pejorative sense, and therefore largely *normatively* null and practically irrelevant. I shall briefly survey each of these lines of criticism in turn, although it should be noted that they can, and have been, elaborated in different ways. Moreover, it is not hard to see how the first line of criticism can either ground or at least ‘leak’ into the second. It is the second line of criticism that is of especial relevance to the subsequent discussion.^3^

However, to begin with the first criticism: this is the contention that liberal moralism is descriptively deficient. At its most superficial, this claim draws on the observation that one would get at best a distorted idea of what politics is actually like, even in societies that come closest to meeting the conditions of liberal moralism, if one had only liberal moralism as one’s guide. (Not, of course, that political theory should necessarily focus only on the specific, locally dominant forms of political practice.) There is, for example, little real appreciation of how political processes and institutions work in practice, nor of the vast array and variety of obstacles that their effective functioning is required to accommodate or overcome. Political institutions tend to be viewed as no more than instruments or mechanisms constructed solely for the purpose of realising antecedent moral principles or political ideals. There is little sense of how they unavoidably need to respond to complex and often conflicting social pressures, including those arising from broader historical determinants, and the motivations of those who inhabit them. Generally, political processes and institutions have only a thin, yet idealised, role, seemingly etiolated, sanitised and excessively moralised in their operation. In this regard, therefore, liberal moralism seems already to have erased from its conception of politics, even in societies like our own, what might reasonably be thought to be some of its most recognisable and familiar features, and perhaps especially those features of the political landscape that make politics the messy, difficult and morally ambiguous activity that it always is.

Developing such thoughts further, though, it is argued that liberal moralism mischaracterises politics in more fundamental ways. For example, the realities of political power seem to be a perpetual embarrassment within a liberal moralist framework. This typically begins with a model of ideally free and equal individuals and, although these are of course acknowledged to be theoretical constructs, it almost necessarily presents the political in a manner that sidelines, for example, the fact that people are always socially embedded in a variety of relationships of power, with a diverse range of commitments, ethical and other, and never equal in anything except a purely formal sense. While these inequalities and social entanglements might be thought of as in some sense contingent to a philosophical concept of the person, they are not, or so the realist argues, contingently related to politics. Politics is always, at least in part but also fundamentally, about the need to manage and accommodate precisely these kinds of features of human life. Furthermore, the fact that it is necessary to have access to political power to achieve almost anything at all in politics, and therefore much of politics is about contesting, pursuing, preserving or enhancing power, is constitutive of politics in pretty much any form. This will present political agents with an extensive range of challenges that are typically passed over by liberal moralists. Even in the relatively benign conditions of an effectively functioning democracy it means at least to some extent courting public opinion, exploiting contingent circumstances and designing policies that can be expected to have widespread popular appeal if one is to win an election, rather than campaigning in accordance with philosophically justified moral principles. The focus of liberal moralism is almost always only on a single, too narrow question: that is, to what ends may political power be legitimately utilised? It singularly fails to address questions about what might be needed to attain political power, and then to maintain it, or even to resist it.

In short, the argument from descriptive inadequacy holds that there is something fundamentally false or misleading in the picture of politics that liberal moralism paints: politics is in many ways significantly different from and perhaps ultimately incompatible with how liberal moralism portrays it. Moreover, this deficiency is not merely trivial or incidental feature of liberal moralism; rather, it is deeply embedded in its assumptions and theoretical practice, and is a failing that seriously undermines its claims to be an appropriate form of *political* theory. This is especially true, as the second criticism makes clear, of a form of political theory that also claims to be speaking to practical political concerns.

The second line of argument against liberal moralism, and the one with which I shall subsequently be most concerned, relates to its alleged normative irrelevance, and to what is perceived to be its utopianism and practical naiveté. As we have seen, in part this, too, can be seen as a function of its descriptive inadequacy, but the point is of independent weight. For here the complaint is that, appearances and all claims to the contrary, liberal moralism can provide us with little normative guidance about how we should act in the real world. Because the idealising assumptions of liberal moralism leave it at some considerable remove from the world as it is (and this should not be taken to imply a simplistic or positivist conception of the nature of political reality), its bearing on how we should act, even were one to accept the validity of its normative principles, becomes at best vague and at worst irrelevant. Again, there are several aspects of this line of complaint, although in what follows I shall mention only a couple.

First, consider, for example, the role that the idea of what he calls ‘strict compliance’ plays in Rawls’s theory of justice. Rawls informs us that within his theory of justice ‘everyone is presumed to act justly and to do his part in upholding just institutions’ (Rawls 1999: 8). However, to ask the question: ‘What would be just assuming that everyone acted justly?’ is, it can be objected, not really to ask a politically relevant question at all; or at least not to ask one with much genuinely normative import. For, whatever the circumstances of politics, they do not include (and presumably never will) anything close to those of the idealising assumption of ‘strict compliance’. Rawls is no doubt aware of all this, but still views strict compliance as entirely consistent with his idea of ‘political philosophy as realistically utopian: that is probing the limits of practicable political possibility’ (Rawls 2001: 4). To realist critics, though, this is more likely to appear plain, unqualified utopianism, well beyond anything that is a practicable political possibility. For one of the ‘facts’ that politics has to engage with is that, whether or not Rawls’s principles of justice are the ‘right’ ones (whatever exactly that may mean), there is not the slightest chance that everyone will in fact agree on them or accept their normative validity. Nor will those who do endorse them at a very high level of abstraction consistently interpret or apply the principles in a similar manner. And even those who do broadly agree at the level of interpretation and application may not in some circumstances be sufficiently motivated to act on those principles, for good or bad reasons. In all these respects, it can be argued, assuming strict compliance renders the normative relevance of Rawls’s theory largely nugatory. For politics has to confront the problems that non-compliance gives rise to, rather than idealise them out of existence.

Mark Philp makes a related point when he argues that although we can imagine a just liberal state, we can never achieve it. This likewise suggests that the issue for us will always be about what to do under non-ideal conditions (Philp 2007). About the latter, in my view, he is certainly right; but I would be inclined to go further and doubt whether many of us can truly imagine a wholly just liberal state, even leaving aside non-ideal conditions. And this is not a failure of imagination. For instance, few people are persuaded by Rawls’s ‘difference principle’ as the basis of a just distribution of economic and other major social goods, but not necessarily because they have a clear and cogent alternative. Rather, the pull that the frequently conflicting claims of distributive principles such as welfare, need, opportunity and desert have for many of us simply do not admit of systematic resolution within a tidy theory. And nor is it at all clear why they should do. For instance, if there is any truth to value-pluralism, it will not necessarily be possible harmoniously to reconcile all the legitimate conflicting distributive values in play in diverse and complex societies like our own.^4^ Nor is there any reason to think that in practice seeking to remedy one injustice may not create or exacerbate another, a familiar enough feature of policy initiatives. But, equally importantly, for many of us what is politically better, which, it should be stressed, does *not* mean ‘ideal’, will usually vary according to context: how wealthy a society is; its social and political culture and operative values; levels of education and literacy; what social, economic and other challenges it faces; the state of public opinion; the external context in which a society has to operate; and much else besides. Thus, although I regard myself (perhaps mistakenly) to be above averagely reflective about such matters, I cannot in all honesty say what I think a just society per se would look like. I can claim to be able to identify at least a few features that (in my view) would be absent from any just society, and about some of these there might be a high degree of consensus. However, even when there is such agreement about particular evils, it need not follow that there will be much agreement about how to weigh them against each other or what to do about them, and not merely for technical reasons, but because different values may be involved in how we try to eradicate even agreed evils. Moreover, it is simply not the case, as Rawls and others sometimes mistakenly claim, that we need to have an idea of a fully just society to be able to identify any particular injustices: indeed, quite the reverse, it is invariably considerably less difficult (and generally less controversial, though certainly not uncontroversial) to identify the latter than it is the former.

One of the most fundamental and enduring lacunae in liberal moralist theorising, and this is the second point, is its lack of any serious engagement with issues of political agency. For, one question that naturally arises in relation to this kind of theorising is: how are the political principles that are endorsed by liberal moralists to be realised? It is important not to misunderstand this question: what is being sought is not what might be called ‘a detailed implementation strategy’ or ‘action plan’, in the language of modern managerialism. The sometimes irritated response that liberal moralists are prone to give – that this is not the business of normative political theory – would have some force if this is what was meant. Rather, the point is that effecting political change is itself, through and through, a political matter: politics is not simply (or at all?) an activity that takes place in a space created for it once we have established a just society. Thus, one might reasonably expect any political theory to have something significant to say about this rather large question. But, liberal moralism is typically either silent on such matters, utterly naïve about them, or effectively seems to require its political principles to be already operative as a condition for realising them. Surely, it is not unreasonable to expect a normative political theory to have something relevant to say about the process of transition by which we get from where we are to where we supposedly ought to be. And this is especially pressing if one takes the view – supported pretty conclusively by the whole of human experience – that in such matters as this one will never arrive at where one ought to be, as politics is always in transition; and still further exacerbated because where one ought to be is always to some degree itself subject to change and political contestation.

So, the upshot of this second line of criticism is that liberal moralism is, contrary to its own self-image, in some important respect normatively irrelevant. And this, I think in two ways that it is useful to distinguish more clearly than I have done so far, although they are also inevitably closely intertwined. These I label the ‘moralist’ and ‘practical’ strands to the normative critique. The moralist strand holds that in making politics subservient to a philosophically constructed moral theory liberal moralism is indeed guilty of a kind of inappropriate moralism. This amounts at the very least to the complaint that in giving primacy to morality in this way, the relationship between morality and politics is importantly misconceived; although it is essential not to interpret this criticism as asserting that politics is entirely independent of morality. Rather, the relationship between morality and politics is more complex than one in which the latter is taken to be no more than an application of the former. The second strand holds that the supposed normativity of liberal moralism is anyway *practically* irrelevant. This links, too, with the descriptive inadequacy charge, because at the heart of this complaint is the thought that, because it is so distant from the reality of politics, not least in neglecting key features of political life, liberal moralism can provide little if any practical guidance as to how we should act in concrete political contexts.

However, rather than explore these critiques further I want at this point to change tack. This involves making a large assumption in accepting that, for the most part at least, there is some substance to the realist critique. No doubt many theorists will immediately conclude from this that the subsequent discussion is based on a false premise. And it should in fairness be noted that there has, as one would expect, been some vigorous counter-argument in defence of liberal moralism, mostly rejecting the realist critique.^5^ Those, though, are arguments that I cannot address here, which is not to deny that at some stage they do need to be addressed. However, even defenders of liberal moralism who believe the realist critique to be of little or no merit, might welcome the thought that the questions raised by the realist critics should be turned back on the realist critics themselves. And, the principal focus in what follows does just that in asking what some of the implications might be for political theory, if one takes the realist critique seriously. More precisely, my concern is with one specific feature of the realist critique: its claim to undermine or deny the normative relevance of liberal moralism. In particular, therefore, I shall be concerned with what better story about normativity a more ‘realist’ political theory might have to tell.

To begin specifically with the charge of ‘moralism’; as we have seen, the realist typically rejects the idea that political theory should simply be understood as a branch of moral philosophy and politics as the practical application of independently conceived moral principles. But rejecting liberal moralism does not tell us how moral normativity and politics are related, as they surely are.^6^ One suggestion for how we might think about this relationship, and which seems promising, is that of Bernard Williams, when he talks of the normative standards of politics arising from the nature of politics itself rather than as something imposed on it from the outside, particularly by an independent moral theory. On this view, the very idea of the political generates its own normative concerns and standards. His point, as I understand it, is that politics has to be understood as a way of dealing with a certain sort of problem – basically the Hobbesian problem of order – which means that politics needs to have at least a minimal normative content, but a normative content that is internal to the activity of politics itself. As Williams puts it:

The situation of one lot of people terrorizing another lot of people is not per se a political situation: it is, rather, the situation which the existence of the political is in the first place supposed to alleviate (replace). If the power of one lot of people over another is to represent a solution to the first political question, and not itself be part of the problem, *something* has to be said to explain (to the less empowered, to concerned bystanders, to children being educated in this structure, etc.) what the difference is between the solution and the problem, and that cannot simply be an account of successful domination (Williams 2005: 5).

For Williams, politics has to be about something more than the exercise of brute force, and that something is to be found in the notion that those subject to the exercise of political power need to be offered some sort of justification for its exercise. Political power seeks legitimacy and that involves giving reasons to those who are subject to it; reasons moreover that amount to more than just ‘because it is my will’ or ‘because I can’.

I am inclined to think that there is something right about this, and that there is an important insight here, not least about the significance of the Hobbesian problematic from which Williams begins. The difficulty, though, is to know how to develop the insight. Williams’s own brief and somewhat tentative attempt to do so is through his idea of the ‘Basic Legitimation Demand’, but this seems to me less of a success. The principal reason for this is that, when he elaborates it, the argument sails perilously close to endorsing the familiar principle of liberal legitimacy, a point that has been well made by Sleat (2010).^7^ And although he appears aware of this danger, Williams did not live long enough to show how it is to be avoided. Better might be to adopt a more minimalist interpretation of what is meant by ‘something has to be said’ to those at the sharp end of the exercise political power, but then the question is what kind of ‘something’; and the worry here is that it may turn out to be either too undemanding or too contestable, as is also the case with minimal moral universalism, which I consider next. However, I am not yet persuaded that we should altogether give up on Williams underlying thought, something which I have tentatively tried to pursue myself, although I must admit without much success, through the idea of a political theory of modus vivendi (Horton 2007, 2010a, b). But, in my view, it may be better suited to a less prescriptively oriented kind of political theory, an issue to which I shall return later.

Another approach, although perhaps one not entirely distinct from that of Williams, is the ‘minimal moral universalism’ of theorists like Gray (2000) and Stuart Hampshire (Hampshire 1999), although Gray for one would probably be unhappy with that label. The idea here is that there are some minimal moral standards – largely conceived negatively as obvious evils to be avoided – that set the boundaries to legitimate politics. But again, although attractive in some respects, and perhaps even in some regard unavoidable, there remain significant problems. First, there is the question of how these values are to be arrived at; and they certainly seem to arise from sources external to politics itself. Thus, Gray, for example, pursues a naturalistic course, looking to biology, evolution and the evidence of human experience, while Hampshire adopts a conventionalist approach, seeking to identify a broad consensus about universal evils. Neither approach, though, is without its difficulties. Secondly, there is the matter of what we should say about the fact that something which looks very much like politics, and many would hold to be ‘legitimate’ politics too, seems easily to co-exist with a failure to observe these standards. For instance, torture is often cited as one such universal evil, but is it really true that, however objectionable, the use of torture is in all contexts *necessarily* incompatible with a broadly legitimate politics? A decision about whether torture may be permissible in some exceptional circumstances looks like a political question. Finally, as with Williams, there seems to be an almost ineluctable tendency for the supposedly ‘minimal’ values to expand, and in the process become more substantial than was initially envisaged. In short, what begin as a few basic ‘evils’ turns out to be a good deal more far-reaching and demanding in their import, once they are fully articulated.

In many discussions of the relationship between political theory and morality the problem is typically presented as one of knowing where a line is to be drawn; a question of avoiding too little or too much ‘morality’. However, it is doubtful that this is really a helpful way of understanding the issues. They are, perhaps, better understood, as Williams does, in terms of *how* morality enters into political theory, rather than *how much* morality a political theory should incorporate. But thinking of the issues in these terms will only take us so far. For, arguably, the key question is ultimately not that either: rather, it is that in so far as normativity does form a part of political theory in what, or from where, does its cognitive or practical authority over political agency derive? Unless there is an adequate answer to this question, then even in its more morally minimalist incarnations it can seem that political theory is no more than another voice in political debate and argument; one that is without any special claim to normative authority. At its sharpest, the question arises of whether political theory is really no more than another manifestation of political ideology (Geuss 2008)? A question that neatly leads to my second set of concerns: those to do with practicality.

This second concern of realists may be approached somewhat indirectly through reflecting on a feature of current British politics, although I do not imagine things are very different in most other countries. So, for example, during the last government we heard much in the media and other public fora about whether the policies of the coalition government were *fair*. From the point of view of political theorists, there are no doubt a number of interesting features of the public discussion of this question, but I want to note only one: this is the fact that although the most famous theory of distributive justice of the last fifty years was styled by its author as ‘justice as fairness’, one will search long and hard to find any reference, even implicitly, to Rawls’s ideas in the endless pages devoted to the coalition policies, not merely in the tabloids but also in the ‘quality’ press and more upmarket magazines. Nor, indeed, can I recollect a reference to any theory of justice or political theorists of note, although various economic theories and economists are mentioned quite frequently. And this is not in the least unusual. Only if a commentary on some aspect of politics is actually written by a political theorist is there a chance that any ideas in political theory might be mentioned, let alone receive serious consideration. People outside of the ‘profession’, not least politicians, simply do not look to political theorists for any sort of guidance about what to do when it comes to either what the goals of political action should be or how to act politically. Such observations may be regarded as superficial, and it is true that explicit reference is not the only measure of influence, but I do not think that they are entirely without some indicative significance.

While not unique, in that politicians often treat expertise high-handedly, this situation is still worthy of note. For, not merely by contrast with economists, lawyers or empirical social scientists, but even by contrast with many areas of applied ethics, such as professional ethics, bioethics, the ethics of sport and so on, the views of political theorists are thought to have no claims at all to any special consideration. Specialists in these other areas are at least thought to have some relevant contribution to make to deliberation about public policy in their appropriate area, even if policy decisions must ultimately be made by governing bodies or political officials. Thus, it would be unusual to find any independent body or official enquiry examining issues related to these subjects without an appropriate professional philosopher (or ‘ethicist’) among their number. By and large, however, politicians and the general public are utterly indifferent to political theory, and do not appear to feel the slightest embarrassment in knowing nothing about it.^8^ And, in truth, this is little more than a mirror image of the attitude many political theorists, and especially liberal moralists, typically seem to have towards politics and politicians, which again seems interestingly different from, say, the views of philosophers of science about science and scientists, or aestheticians towards art and artists.

What should we to make of this? Some political theorists certainly believe that political theory ought to be useful and should actually be used for practical guidance; and that therefore something is amiss if this is not happening. To simplify greatly, it is a fairly common view, especially among liberal moralists and ideal theorists generally, that political theory should articulate the ideal (of justice for example) while political action should be about trying to realise it; although this may be no easy matter and may not be an area in which political theorists have much to contribute. But it is precisely this kind of picture of the relationship between political theory and practice that is characteristically rejected by realists. For them, the detachment of a political ideal from actual political experience is one of the symptoms of what is wrong with liberal moralism. But if that is the problem, what is their alternative? Interestingly, on this point there is a notable schism among realists about what is a better way of thinking about the relationship between political theory, morality and practice.

On the one hand, there is a view that the main point of a more realistic political theory *is* to make it more practically relevant, and less in thrall to an impossibly high-minded moralism that is politically merely utopian. And perhaps the arch realist in the history of political theory, Machiavelli, could be cited in support of this view. No doubt among other things, Machiavelli (at least in *The Prince*) was clearly interested in offering practical advice to political rulers. On this view, a realist political theory, while not of course Machiavellian in the now colloquial sense, and not necessarily directed exclusively to political leaders rather than citizens, is about developing a more practically relevant kind of theorising than the unworldly utopianism of ideal theory associated with liberal moralism. It should aim to be more grounded in the realities of political life, with a view not to eschewing being practically useful, but to being rather more effectively so. For these realists, the principal reason for the failure of political theory to achieve greater practical salience lies, at least in large part, in the character of liberal moralism, which renders itself practically irrelevant. What is needed on this view, therefore, is less ideal theorising, abstracting from theorising the factors that make politics the messy business that it is, and instead a greater willingness to engage with the ethical and practical complexities of political action in the ‘real world’ in a way that would be conducive to making political theory more genuinely and appropriately action-guiding. Naturally, they sometimes have different views about precisely how this should be pursued, but the aim and the critique are broadly shared. Adopting some useful terminology from Michael Freeden, these theorists can be labelled ‘prescriptive realists’ (Freeden 2012).

While sympathetic to the critique of the practical relevance of liberal moralism advanced by such prescriptive realists, this is not the positive account of the role of a more realist political theory that I want to endorse. Rather, my inclinations lie with another camp of realists, who are labelled by Freeden ‘interpretative realists’. On this view, it is not really the business of political theory to be offering guidance or advice to politicians, political activists or even citizens in general. Rather, what we should learn from a realist orientation is that the activity of politics is for those practically engaged in it, whereas political theory, by contrast, should not be engaged in an ideological competition, but be primarily about trying to understand, to make sense of politics. This will involve some element of evaluation, as such a political theory is not ‘merely descriptive’, in the typically dismissive characterisation of it, a point to which I shall briefly return towards the end. However, it involves no aspiration to guide political action. To some, this may seem a surprising conclusion for a realist to arrive at.

Let me elaborate just a little, therefore, by returning for a moment to the fairness (or otherwise) of the coalition government’s policies: on the interpretative view, political theory can elucidate and analyse the conceptions of fairness that are in play, explore their coherence, presuppositions, implications, supporting arguments and so on, how they figure in practical political discourse, how they relate to other values and beliefs, and even propose alternative conceptions. Yet it has no particular claim to be able to authoritatively determine what *is* fair, either realistically or ideally. That is: there is no good reason to think that political theory has any normative authority in such matters. There is no good reason to believe that the values favoured by political theorists, or their preference for any particular understanding of those values, has any kind of claim to primacy over those advanced by anyone else. But, this may not quite mean, in Wittgenstein’s famous phrase, ‘leaving everything as it is’. For once, say, these features of different conceptions of fairness have been laid bare this may indeed feed back into political debate and discussion, becoming part of the substantive political process. But whether and how it does, and, if it does, what responses it will elicit is something that will be properly determined by political agents, rather than by political theorists; or, if it is by political theorists, then only when they are acting as political agents rather than as theorists.

Moreover, not only should political theory not be expected to authoritatively determine what is just, it should not either aim to prescribe any particular political actions or institutions. Once more, it can help us see the complexities and obstacles involved, articulate the values that are at stake or inform different possibilities and explore these matters from a variety of perspectives. And in doing so it may also clarify some issues for political agents, but again what use political agents make of it is a matter of their political judgement, and in particular will involve a practical, contextually-sensitive assessment of many factors, and crucially what is politically possible. A political theorist may have opinions about such matters, too, but those opinions again have no special claims to guide or judge political practice. Their opinions may be more or less shrewd, but they have no cognitive or practical authority deriving from their being the judgements of political theorists.

So, what I am suggesting is that seeking to be ‘practically relevant’ need not lie at the heart of a more realist political theory, and should not be a criterion by which its adequacy is judged. In this regard, my proposal is more radical than that of prescriptive realists in seeking a more fundamental reconsideration of our understanding of the point of political theory. All realists will object to the utopianism of ideal theorists purporting to offer practically relevant political guidance. Prescriptive realists respond by trying to offer better guidance; by undertaking a broadly similar task but with greater attention to what they take to be the realities of political life. By contrast, my suggestion is that as interpretative realists we should pretty much give up altogether on the aspiration to be practically politically relevant in this way. Although, as I have just explained, this is not the same as saying that a realist political theory cannot in fact have any practical influence; but whether or not it does will be contingent and, crucially, incidental to its purpose. And if it does have no influence on political practice, that is a situation that as theorists we should embrace with equanimity, and is not a cause for regret or something to lament as a failure. Many theorists, including both liberal moralists and prescriptive realists, will likely see this as a form of mere academicism, a way of trivialising or betraying the historic mission of political theory, of rendering it effectively pointless. But why should this be so? There is no reason why a kind of political theory that aims at understanding and making sense of our political predicament and the ways in which we think about it, including of course the meaning and role of political values, and which tries to reflect on the tensions, ambiguities and complexities to which political life gives rise, should be thought to be insignificant or without value. Furthermore, it remains genuinely political in the sense that it makes politics its *subject*, but not in the sense that it is *politically motivated*. Interpretative realism has no ideological axe to grind.

What then might a more realistic political theory look like? Perhaps surprisingly, given my claim for its radical contentions, in many respects it may not look so *very* different from some aspects of the political theory we are familiar with, in that it will also be concerned with many of the values that dominate liberal moralism. But, the approach will be different. Crucially, as far as normativity is concerned – the issue on which I have focused here – it will aim to be neither ideal nor prescriptive. And not being ideal, it will not engage in the extensive processes of idealisation that typically characterise liberal moralism. Rather, it will be much more attentive to the circumstantial and contingent character of politics and to the kinds of conditions and constraints under which it is variously practiced. It will not, for example, assume perfect compliance but accept that any political arrangements must have at their centre the recognition that one of the things with which politics must always deal is non-compliance and different views about the appropriateness of compliance on any particular terms. It will accept that disagreement extends not only to conceptions of the good life, but also to principles of justice, and that politics has to find ways to accommodate that level of disagreement, too. However, it is not the role of political theory to resolve such disputes, but to understand them. Moreover, such a theory will likely be less obsessed with a handful of principles; rather it should be receptive to other issues, such as the place of leadership, the role of contingency, the idea of political judgement and the meaning of political possibility, which are all issues that are largely neglected within the paradigm of liberal moralism.

It will also eschew prescription in the straightforward sense that it will not have being action-guiding as one of its principal aspirations. Instead, it will aim, for example, at trying to understand the fundamental concepts of political discourse and argument and at elucidating the structures of different ways of thinking about politics. This may well have implications that bear on political practice, and then again it may not, and even when it does they may be opaque and unpredictable; but crucially this is not the point of the enquiry. One, slightly fanciful, way of making this point is to say that political theory is neither motivated by the desire to critique nor by the desire to reconcile us with reality, neither with Marx nor with Hegel, but potentially both: it is at root cognitively motivated by the desire to understand. But, of course, understanding is an aspiration, and it can fail in a number of ways and for a number of different reasons. Such a failure may fuel discontent, but need not; any more than the achievement of some understanding will necessarily lead to quiescence. And nor is there any reason to think any given understanding will command universal assent, rather than at best being a platform for a different, and potentially better, understanding.

The picture of a realist political theory that I want to present is one that is more recognisably about politics as we know it, and in that sense is indeed genuinely more realistic, but it does not seek to be a kind of *ersatz* politics itself, and in that respect it remains theoretical. In fact, elements and examples of this can be found within the contemporary practice of political theory without too much difficulty, but too often they are subservient to the idealising and prescriptive tendencies that interpretative realists want to jettison. It is an image of political theory as reflective and existing, to borrow a phrase, ‘in a cool place’ (Phillips 1999). And that coolness lies in the detachment of political theory from political engagement, not in various forms of idealisation. So, we can continue to enjoy *The West Wing* for what it is – largely escapism and wish-fulfilment, leavened with occasional perceptiveness – but let us not model political theory on it.

Yet there remains at least one troubling issue for the kind of political theory I advocate to address, and one which represents a challenge to the claims I have been making for it. It is implausible to think that political theory can entirely escape all normative commitments. And this is so for at least three reasons. First, those thinkers, such as Michael Oakeshott, who have apparently aspired to such a conception of political philosophy, have fairly obviously failed. Moreover, even conceptual analysis typically cannot entirely avoid at least some implicit, if only minimal, normative commitments. Second, politics is itself inextricably bound up with the normative in a way that makes it seemingly impossible for the theorist to be *entirely* disengaged from some degree of evaluation of the normative claims that are part of politics. One cannot, so to speak, stand outside or above absolutely all normative claims: there is no place which is *that* cool. And third, even a political theory devoted much more to *understanding* will, as has already been mentioned, be marked by normative inflections of various kinds. For instance, what we take to be fundamental to politics will unavoidably contain within it some ideas, however modest, about how it should be pursued, ideas about better and worse, political success and failure. So, even an account of political theory that eschews normativity as its aim cannot entirely escape some element of normativity in practice. But, while this is something that any fuller account of a more realistic political theory along the lines I have merely sketched here will have to come to grips with, I do not think that this difficulty undermines the distinction between a political theory that aims at understanding and one *motivated* by political prescription or normativity more generally.

It should go without saying that much of the discussion here has been broad-brush and its arguments often little more than gestural: it may be read as no more than an invitation to engage with a different way of thinking about political theory. Moreover, as just noted, there remains an important unanswered question about the ineliminably normative dimension of even a non-normatively driven and interpretatively oriented political theory. And while there are of course many other issues to which the realist critique gives rise that are also important, and with which I have not engaged here, I think that questions about normativity lie at the heart of that critique, and are among the most perplexing for liberal moralists and their realist critics alike. But that is no more than an invitation to think more deeply about what a more realistic political theory might look like.^9^

## Footnotes

1 I make no distinction in the subsequent discussion between political theory and political philosophy

2 See, for example: Dunn 1985; Shklar 1989; Honig 1993; Hampshire 1999; Gray 2000; Newey 2001; Williams 2005; Mouffe 2005; Philp 2007; Geuss 2008; Sleat, 2013. For a more sceptical, but not entirely dismissive, evaluation of the realist critique, see C. A. J. Coady 2008.

3 The subsequent paragraphs elucidating the realist critique of liberal moralism draw extensively on Horton 2010a.

4 On value pluralism see Gray 2000.

5 See, for example: Swift 2008; Stemplowska 2008; Simmons 2010.

6 Raymond Geuss seems to be the only realist of whom I am aware who might dissent from this view, but I find him neither consistent nor especially plausible in this regard. It seems to me that he grossly underestimates the importance of ‘beliefs’, which in some form or other are, after all, what ultimately motivate political agents to action (Stemplowska 2008).

7 For an interesting and rather more sympathetic reading of Williams on this point, see Hall 2015.

8 It should perhaps be noted that think tanks and policy advisors have occasionally been known to ransack political theory for ideas. This has usually taken the form of seeking rhetorical justifications for positions that are already held or drawing on much lower level theory than liberal moralism for ‘bright ideas’.

9 Earlier versions of this article were presented as the Alan Milne Annual Memorial Lecture at the University of Durham in November, 2011 and as the Jean-Jacques Rousseau Lecture at Keele University in February, 2012. I am grateful to Maria Dimova-Cookson and Sorin Baiasu respectively for inviting me, to the audiences on both occasions for the very helpful discussions, and to Margaret Canovan and three anonymous referees for this journal for their mostly sympathetic yet perceptive written comments.
